# COVID-Pandemic in Nepal: An Opportunity to Institutionalise Local Governance in School Education

**DOI:** 10.1177/00345237231160079

**Published:** 2023-03-12

**Authors:** Devendra Adhikari, Dr Chandra Sharma Poudyal, Dr Rajan Binayek Pasa

**Affiliations:** School of Education, 20204631 University of Waikato, Hamilton, New Zealand; Universal College of Learning(UCOL):Te Pūkenga, Manawatū, New Zealand; Central Deparment of Rural Development, 2097983 Tribhuvan University, Nepal

**Keywords:** COVID-19, local governments, school governance, SMC

## Abstract

The 2015 constitution of Nepal declared Nepal to be a Federal, Democratic, and Republic nation. The constitution has provided local governments the right to manage school education, and the 2017 Local Government Operation Act of Nepal has outlined the roles and responsibilities of local governments in taking guardianship of school education. Since 2017, Nepal has been in the process of institutionalising decentralised governance practices, but this is proceeding at a slow pace. Unprecedently, from early 2020, the COVID-pandemic hit hard the lives of Nepali people, and Nepali education suffered greatly. The pandemic has brought several opportunities for the government to devolve the management system of school education to local levels. This article is based upon the analysis of the six newly introduced plans, regulations, and guidelines in Nepali education, that aim to continue school education amid the COVID-pandemic. These newly introduced polices have significantly acknowledged and mobilised the local strengths in educational management. This research supports a new academic discussion on governance ideas of the Nepalese education system, induced by the COVID- 19 pandemic.

## Introduction

The modern education system began after the downfall of the autocratic Rana regime in 1951. The political system until 1990 was not fully democratic; thereby, educational development was poor and highly centralised (Parajuli, 2019). The 1990 People’s revolution for democracy was successful in overthrowing the authoritarian reign of the king and bringing about a new era of development. The education system began to advance after that time. In 1992, the government formed the National Education Commission after realising that “the inspection system had to be re-built from the bottom up if it was to play its proper role in the qualitative improvement of national education"(Nepal National Education Commission, 1992: 70). Henceforth, the country felt it necessary to develop a bottom-up governing system in public schools across the country. The discussion that follows concerns the decentralisation efforts of the government of Nepal at different points of time.

## Decentralising School Governance in Nepal: 1990 to 2019

Nepal became more progressive towards education development in early 2000. The seventh amendment of the 1971 Education Act in 2001 recognised local governance as an important stakeholder in school education (Nepal Ministry of Law, Justice and Parliamentary Affairs, 2017b). In 2003, with the support from donor communities, Nepal initiated the expansion of the School Management Committee (SMC) model in all public schools across the country. A SMC comprises parents, donors, educators, founders, local intellectuals, elected ward representatives, teachers’ representative, and head teacher. This nine membered committee has at least three women. SMC members are voluntary positions created to fulfil government legal formalities only, and they play an advisory role (Nepal Ministry of Law, 2017). Decentralised school governance through SMCs helps increase the enrolment rate, thus reducing the gender disparities in schools and beginning the lifelong teaching-learning process (Nepal National Planning Commission, 2016). The interest of parents in school activities increased and they started to raise local funds for schools. Locals became aware of the different activities in schools and parents started showing more concern over their children’s learning outcomes. Teachers’ accountability in terms of their professional role and regular attendance has increased due to the close monitoring by parents and communities (The World Bank, 2010). SMCs have ensured the representation of women who tend to promote the participatory development practices at the community level (Laurent-Olive and Bourn, 2020). These advancements indicate that the handing over of responsibilities for school management to communities is helpful in promoting school education in Nepal.

In addition, various educational plans that acknowledge the local strengths in school management were developed and implemented in Nepal in early 2000. Education for All (2001–2015) stated that “ownership of governance of basic education will be provided to communities, through appropriate representation in the form of empowered SMCs and Parent-Teacher Associations” (Nepal Ministry of Education and Sports, 2003: 32). Likewise, the School Sector Reform Plan (2009–2015) delineated the roles of SMCs in being responsible to enhance the community and parents’ support to schools by developing the duo’s interest and ownership. The adoption of the School Sector Reform Plan in 2009 was a significant step for Nepal in recognising the extensive roles of communities in managing public schools (Nepal Ministry of Education, 2009). The School Sector Development Plan (SSDP: 2016–2023) has focused on enhancing the inclusive access to school education by reducing disparities; building the infrastructure destroyed by the devastating earthquake in 2015; setting up the educational administrative structure as per the new federal structure of the country; and delineating more educational authority to the local governments (Nepal Ministry of Education, 2016). Thus, the Government’s motives have been to streamline the education development process through the decentralisation of school management systems. (Shrestha, n. d)

After the promulgation of the 2015 constitution, the centralised governance system in Nepal has been delegated to seven provincial, and 753 local governments. Public education has been listed among the concurrent responsibilities of three levels of government in Nepal (Nepal Ministry of Law, Justice and Parliamentary Affairs, 2015). The 2017 Local Government Operation Act has provided enormous authority to local governments in managing basic and secondary education (Nepal Ministry of Law, Justice and Parliamentary Affairs, 2017a). The 1971 Education Act (amended in 2017) stipulates that every public school in Nepal shall have SMCs to “operate, supervise, and manage” the school education (Nepal Ministry of Law, Justice and Parliamentary Affairs, 2017b: 30). The 2019 Nepal National Education Policy, approved by the government of Nepal, mentioned that SMCs will be redefined as the supporting mechanisms of the local governments in reforming school education in its territory [clause 10.34.4] (Nepal Ministry of Education, Science and Technology, 2019a). The 2020 Nepal’s 15th Plan has acknowledged the roles of all levels of governments, political parties, local intellectuals, social activists, and government and non-government organizations crucial in increasing the enrolment of all children in schools (Nepal National Planning Commission, 2020a). After 2017, some of the local government policies include the leadership of elected representatives as SMC chairpersons. The 2020 Education Policy of Lalitpur Metropolitan City, Nepal specifies that every public school in Lalitpur Metro Politian City will have SMCs to support the school management work. These SMCs will be chaired either by a ward chairperson or any of the other elected ward committee members in the city (Nepal Lalitpur Metropolitan City, 2020). These new policy approaches indicate school governance authority is being divested to local levels in Nepal.

In summary, the year 2015 induced aspirations of change in the lives of the Nepali people. Every political regime imposed its visions and policies on the socio-economic lives of people, including in education (Sharma Poudyal, 2013). The political stability in the country, of course, was the reason behind the interventions in the development of the education sector. After 2015 to early 2020, there were introduction of eight different regulations, policy, and plans pertaining to the decentralisation of school education from the central to the local level. This shows the gradual education decentralisation process to local governments in Nepal. The summary is presented in the below Table 1.

**Table 1.** Summary of educational related policy documents and guidelines before the COVID-pandemic.

S.n	Government bylaws	School governance through local governments
1	The 2015 constitution of Nepal	Rights of local governments in managing school education
2	School sector development plan, 2016–2023	Importance of local governments in school education
3	The 2017 local government operation Act	Roles and responsibilities of local governments
4	The 1971 education Act (amended in 2017)	Procedures to form SMC and the selection of SMC chair
5	The 2019 national education policy^1^	Acknowledgment of local governments and legitimisation of their authority in school governance
6	Sustainable development goals 2016–2030	Quality education through a capacitated team
7	The 2020 Nepal fifteenth plan	Acknowledgment of the strength of localism in school governance
8	Local education acts [of each local government]	Locally elected representatives, on chairing the SMCs: The 2020 local education Act of lalitpur metropolitan city

## (Deteriorated?) public education in COVID times

Up to 2019, there had been nearly two decades of community engagement in school management in Nepal. Although there have been some notable improvements in educational development from macro-educational perspectives (The World Bank, 2010; Nepal National Planning Commission, 2016), some research suggests that the academic outputs of the government schools in Nepal have not significantly improved (Joshi, 2018; Nepal Ministry of Education, Science and Technology, 2020f; Koirala, 2015). The faulty decentralisation process and inefficient School Management Committees are likely to be responsible for poor education outputs (Carney et al., 2007; Pherali, 2012). There is also a claim that the education system has never been fully decentralised in Nepal (Hamal, 2020). These criticisms indicate that the decentralisation of education in Nepal is a herculean task which is unable to enhance public education.

When the COVID-pandemic began in March 2020 in Nepal, all sectors including education sector were negatively impacted. Schools were closed for several months (United Nations Children’s Fund, 2021b). Until now, the country has witnessed three waves of the COVID-pandemic. The first wave was from March to September 2020, the second wave^2^ was from April-August 2021, and the third wave^3^ was from January- February 2022. The schools were physically closed almost all the time in the first and second waves, and for a month during the third wave. Millions of children were out of schools in Nepal due to the pandemic. Virtual classes were conducted, but these remained mostly ineffective because this was a piloting period from the conventional pedagogical system to IT-based learning. Most users were not experienced with the learning-based technologies, and the lack of internet connectivity in most of the rural regions in Nepal added to this already disadvantaging situation (Paudel, 2021; Surkhali and Garbuja, 2020). During the COVID period, the digital divide in the education system was severe in Nepal (United Nations Development Programme, 2020), so the school education was seriously obstructed during the COVID-pandemic in Nepal (Katyal and Charlton, 2021; Dawadi et al., 2020).

During the pandemic period, the Government of Nepal issued and implemented six different educational plans, regulations, and guidelines [discussed below] to ease the situation. In the short run, these guidelines addressed problems such as continuing teaching-learning through alternative methods and implementing needful approaches for school management. As a consequence, these guidelines had a large impact on institutionalising the local governance practices in school education. In the guidelines, there are many indicators to local governments to take responsibility for crisis-handling in school education. These new interventions might help the Central Government in educational power-sharing with local governments as the “pandemic has created an opportunity for the state to accelerate the federalisation process” (Karki, 2020: 4). For this reason, it is important to unpack what these guidelines mean and how these could strengthen the local roles in school education.

## Local governance perspectives in school education

Local government is the extended form of the central and provincial government, formed to devolve specific rights and services of people at their doorsteps. In this regard, local governance is a wider perspective, where communities have collective decision-making authority to choose the accountable local leadership themselves. The idea of local governance is also aimed at ensuring the democratic participation of local people in any development that helps to improve their quality of life (Shah, 2006). Local governance mutually benefits from the vertical political system and from the horizontal dimension that includes the community and the market (Keuffer and Horber-Papazian, 2020; Kersbergen and Waarden, 2004). Moreover, local governance makes citizens more responsible and establishes their roles as “decision makers and beneficiaries of public policies” (Casula, 2017: 1134). Thus, the local governance mechanism is there for the benefit of the people.

Despite decentralisation being a good practice that devolves power to local authorities, there may exist conflict in terms of power-sharing and resources. This situation might create conflict among different tiers of government which in turn results in complexity in intergovernmental collaboration (Steiner et al., 2018). Accountability of local governments can be increased if they are allowed to perform their duties without undue interference from the central authorities (Andrew and Goldsmith, 1998).

The worldwide development agendas through Sustainable Development Goals (SDGs) by the United Nations have kept the ‘glocalisation’ as its core concept. It involves connecting the locals with the rest world, and prioritising the locally managed institutions, local economies and local participation in development efforts. Macro-level development is dependent upon the mobilisation and meaningful progress of grassroots institutions. The issue of localism in development is for, “grounding development choices in the will of the people through popular participation and ownership” (United Nations Development Programme, 2016: vii). In the most recent decade, the concern of local governance has been more aligned towards prioritising public welfare (Stoker, 2011), including quality education delivery.

In the context of the education system in Nepal, the concept of local governance has been influential in recent times. The political changes over different timeframes in Nepal has brought arguments on several educational reforms over the 1990s to 2000; the bottom-up school management process (Nepal National Education Commission, 1992), 2000–2015; SMCs being recognised as having an important stake of school governance (Nepal Law Commission, 2001; Nepal Ministry of Education, 2009), 2015–2019; constitutional acknowledgment of the school education as of local governments (Nepal Ministry of Law, Justice and Parliamentary Affairs, 2015; Nepal Ministry of Law, Justice and Parliamentary Affairs, 2017a): and in 2020, COVID situation and laws and policies to strengthen local governance in school education (Nepal Ministry of Education, Science and Technology, 2021). This indicates that school governance is shifting towards more local authority.

Before the COVID-pandemic, the Government of Nepal made several efforts concerning educational development through delegation of school governance to the local level. From the constitutional provisioning to Acts and policies, every document highlighted the roles of local levels in developing school education. The global development goals such as SDGs and donor-funded plans such as School Sector Development Plan also stressed localism to develop school education. However, these legal guidelines, plans, and policies were difficult to implement quickly; as it takes time for any interventions to institutionalise in a bureaucratic system (Wildavsky, 1972; Jones et al., 2017; Khanal, 2010). Hamal (2020) stressed that there is no real decentralisation of school education as the central political leadership and bureaucracy do not wish to delegate this authority to subordinate bodies. Bhattarai (2022) claimed that the Central Government does not provide sufficient funding to efficiently administer and develop school education. The Central Government appears unwilling to finalise and implement the Federal Education Act until the sixth year of promulgating the 2015 Constitution (Nepal Ministry of Education, 2021). This act is essential to outline the roles and responsibilities of the Central, Provincial, and Local Governments to promote schooling education. Amid this distressful power devolution situation, we found that the COVID-pandemic eased the institutionalisation process of decentralisation in school governance with new innovations in educational management.

All in all, there is no doubt that local participation and ownership can only preserve and promote good governance practices at local levels and overall in the country’s political scenario (Chaudhary, 2019a). The handling of the COVID-19 pandemic in participatory way through larger consensus amongst all stakeholders can lead towards sustainable results. The positive outcome of applying good governance principles by a political system in crisis has been discussed by United Nation Development Programme (2011). COVID has induced an educational crisis in Nepal that demands extensive collaboration amongst all stakeholders. Democratic norms and good governance are complementary as both aim to yield a stable political system that is responsible and accountable to the people (Dahal, 2017). This could be a reason the Government of Nepal has developed several educational guidelines to strengthen schools and local governments to properly manage the school systems. Hence, local governance theories explain the idea of devolution of power to local governments in school governance in Nepal during the COVID period.

## Research Methods

This article is based upon the review of six plans, frameworks and guidelines (henceforth called policy) prepared by the Government of Nepal to cope with the COVID in school education. Documents analysis help unpack the different educational policies advancements, which are prepared alongside the continuum of time and political necessities (Bowen, 2009). We did a policy document analysis, as suggested by (Cardno, 2018), to analyse six education policies prepared by the Nepal Government after the COVID-pandemic began in 2020. Our focus was on identifying how the authorities delegated to local governments to continue teaching-learning activities helped to strengthen education decentralisation. We assessed the policies and listed the roles of local governments in schooling education during the COVID-pandemic. These new policies by the Nepal Ministry of Education included i) COVID-19 Education Cluster Contingency Plan, 2020; ii) Alternative System’s Learning Facilitation Guidelines, 2020, which was later revised as the Student Learning Facilitation Guidelines, 2020 iii) Secondary Education Examination’s Student Assessment, Result Publication, and Verification Regulations, 2020; iv) School Level Curriculum Adjustment Model, 2020; v) Education Related Emergency Guidelines, 2020; and vi) COVID Related School Operation Guidelines, 2020. The results of this policy document analysis are discussed next using the topic and sub-topics.

## Analysis and discussions

The analysis of the policy documents has expanded to clarify about the different interventions of the Government of Nepal to continue school education amidst the chaos of the COVID-pandemic. The focus of these discussions is the role of the Central government, local governments, SMCs, and schools in managing the education system during the COVID crisis time. The discussion is presented using a main heading COVID-pandemic: the year 2020 in Nepali education, with sub headings on the six new educational politics introduced in the COVID context.

### COVID-pandemic: The year 2020 in Nepali Education

The pandemic situation that occurred in Nepal in 2020 was unforeseen by the education policy makers in Nepal. The nationwide COVID-induced lockdown was announced by the government on 24 March 2020, and all the educational institutions were physically shut down. The situation was very terrible in the country as many people were falling sick and dying due to the virus. Although the priority of the Central Government was the health concerns of Nepali citizens, it tried to re-establish educational activities through wide consultation and global practice. Different educational policies were immediately implemented to normalise the school education system. These new policies included: i) COVID-19 Education Cluster Contingency Plan, 2020; ii) Alternative System’s Learning Facilitation Guidelines, 2020, which was later revised as the Student Learning Facilitation Guidelines, 2020; iii) Secondary Education Examination’s Student Assessment, Result Publication, and Verification Regulations 2020; iv) School Level Curriculum Adjustment Model 2020; v) Education Related Emergency Guidelines 2020, and vi) COVID Related School Operation Guidelines 2020. As follow, we focus on the findings related to the roles of local governments in these six policies.

#### COVID-19 Education Cluster Contingency Plan, 2020

First, an education-related contingency plan was prepared by the Central Government of Nepal as an immediate reflex action against the pandemic. It was approved in May 2020. This plan focused on continuing the education of students and hinted at the promotion of e-learning for students. The plan included the following commitment of the Central Government to resolve the pandemic crisis in school education. It mentioned:

“Ensuring adequate capacity for management and coordination during the response period […] Strengthening prevention and resilience within the school system and among students, teachers, parents and caregivers […] Support continuity of education/learning for all children in all areas including children with disabilities and from marginalized backgrounds”

(Nepal Ministry of Education, Science and Technology, 2020e: 6)

This plan envisaged dealing with the pandemic via a collaborative approach. The Central government sought help from all education stakeholders to deal with the crisis. The plan laid the foundation for initiating virtual learning activities in Nepali schools.

#### Alternative System’s learning facilitation guidelines, 2020

The pandemic situation was becoming worse in the nation after March 2020. There was less chance of physical opening of the schools. Then the Central Government did wide consultation with education experts and implemented the alternative learning facilitation guidelines. It was approved in May 2020. These guidelines were solely intended to continue the teaching-learning process through different means. Almost all the following responsibilities were given to local governments to cope with the pandemic havoc in school education.

a. Identifying and classifying students in the vicinity
b. Establishing the needed learning centres
c. Mobilising teachers as teaching facilitators and evaluators
d. Producing and broadcasting the learning facilitation program through the participation of teachers and students through radio, TV, online, and offline media
e. Awareness campaign regarding digital and distance education, and COVID or similar pandemic
f. Mobilising the locally settled teachers and volunteers as learning facilitators
g. Printing the educational materials produced by central, federal, and local governments and publishing those as books or exercise books for schools
h. Using old books, as necessary
i. Facilitating the provision books to the students in their homes
j. Formulating programs to the categorised groups through ward offices.
k. Empowering teachers to conduct offline or online classes
l. Conducting learning programs through social distancing
m. Receiving assistance from different organisations to conduct programs

(Nepal Ministry of Education, Science and Technology, 2020a: 8–9)

*Student Learning Facilitation Guidelines, 2020.* In September 2020, the Central Government revised the Alternative Learning Facilitation Guidelines. The additional responsibilities of local governments were:

a. Adjusting curriculum and facilitating implementation of the guidelines and other needed works
b. Monitoring the effective implementation of the learning facilitation activities and working on needed facilitative and reformative measures
c. Establishing parental education programs to orientate parents about learning facilitation activities
d. Arranging the necessary resources for differently-abled students, or those who lack facilities.

(Nepal Ministry of Education, Science and Technology, 2020d: 13)

The Nepal Government prepared the guidelines hoping that each local government would act responsibly. The local governments were given these tasks as per their constitutional responsibility toward school education. Most of these governments followed the instructions of the Central Government and continued to facilitate the teaching-learning process in schools. Alternative education through multiple delivery processes (Katyal and Charlton, 2021) was necessary because virtual learning through the internet was not accessible throughout the county (Surkhali and Garbuja, 2020). This guideline acts as a benchmark for formally beginning the alternative education process in Nepal.

#### Secondary Education Examination’s Student Assessment, Result Publication, and Verification Regulations 2020

Every year, several hundred thousand students appear for the Secondary Education Examination (SEE) in Nepal. The exam is conducted for tenth-grade students. In 2020 (2076/7 B.S.^4^), due to the COVID-pandemic, students could not sit for the SEE exam, even though 482,786 students applied (Nepali Sansar, 2020; August 18). Usually, the exam would happen in March-April, but the pandemic obstructed its conduct. As a consequence, the Government prepared the secondary education examination’s student assessment, result publication, and verification regulations in June 2020. The regulations included:

• Every school would internally evaluate the students.
• There shall be a responsible committee formed comprising a school Head Teacher, a class teacher, and a teacher representative, preferably the oldest recruited. This committee would finally approve the evaluation result of the students and send it to Education Development and Coordination Unit (former District Education Office) for the further process.
• The evaluation result shall be forwarded to National Examination Board for the result publication.

(Nepal National Examination Board, 2020: 1–3)

The physical exam was cancelled and the internal evaluation of SEE students was done for the first time in Nepal. This practice also legitimises and emphasises the role of schools in the internal evaluation of their students.

#### School Level Curriculum Adjustment Framework, 2020

In normal times, the academic year of schools in Nepal is from April to March. After September 2020, the COVID restrictions were gradually eased as the COVID cases started dropping. Most of the schools in Nepal were closed for almost 6 months during the beginning of 2020 (2076 B.S.), and online teaching in its rudimentary form was introduced for 6 months from April to September. We referred to this period in the education history of Nepal as COVID holiday. Then in September 2020, the Central Government issued a framework to adjust the school-level curriculum. This revision shortens the curriculum contents, eases the student evaluation process, and facilitates the learning as per local needs and availabilities. This framework includes the following responsibility to local governments:

• Coordinating in the implementation of the Student Learning Facilitation Guide, 2077
• Forming a committee amongst a school principal, a subject teacher, and a local expert in coordination with Education Officer^5^ to implement a revised curriculum. This committee shall coordinate learning facilitation, student evaluation, education accreditation, and learning record and certification
• Counselling and suggesting feedback to the School Management Committee and Principal regarding the continuity of student’s learning
• Facilitating uniform student evaluation amongst all schools in the local government unit
• Providing counselling to parents through ward offices to continue the education of students
• Managing textbooks, exercise books, resource materials for students

(Nepal Ministry of Education, Science and Technology, 2020g: 63)

Schools were given authority to implement the revised curriculum and continue education of students. Local authorities were given permission to implement the revised curriculum and locally-suitable evaluation procedures.

#### Education Related Emergency Guidelines, 2020

In September 2020, the Education Related Emergency Guidelines was prepared by the Government of Nepal. This guideline contained several approaches that would continue the education of students through alternative means. Most of the following responsibilities were given to the schools and local governments.

a. Collecting data: i) Of the migrant students and school changing students (including those who are promoted to upper grades), and ii) Students having access to alternative learning
b. Assuring that all the students received textbooks
c. Continuing students’ enrolment in schools
d. Facilitating teachers’ mobilisation to continue teaching
e. Developing and printing the necessary learning materials
f. Developing a Closed User group among teachers and students at public schools through mobile phone services to enhance learning connectivity
g. Expanding internet services at schools
h. Implementing the concept of ‘Home Schooling’ to engage the students through different learning practices in their homes
i. Establishing the temporary learning facilitation centres
j. Equipping the Learning Facilitation centres with free Wi-Fi zones
k. Ensuring the participation of girls in the learning process
l. Introducing targeted programs to vulnerable groups who have learning problems
m. Revisiting the tuition fees of private schools
n. Coordinating, monitoring, and evaluating school education with other tiers of governments
o. Leading a local educational campaign for the successful implementation of this guideline

(Nepal Ministry of Education, Science and Technology, 2020c: 1–10)

These activities were primarily focused on tackling the impact of COVID on school education. As a consequence, the responsibilities handed to schools and local governments helped to strengthen their capacity to take guardianship on education

#### COVID Related School Operation Guidelines, 2020

Finally, in November 2020, a policy named COVID-related School Operation Guidelines, was launched to physically resume school education. It delegated authority to local governments to decide whether to open schools or not. It included:

The local governments and schools have to work out preparing the bases to re-open the schools, student admission, school operation, teaching-learning management, and postponed exams.

(Nepal Ministry of Education, Science and Technology, 2020b: 2)

The local governments then started consulting with other school stakeholders and school administrations to resume the face-to-face classes. Depending upon the intensity of the COVID-pandemic, schools gradually resumed physical classes in Nepal (Dhungana et al., 2020), and many parents were excited to send their wards to schools after the normalisation of the pandemic (United Nations Children’s Fund, 2021a).

The six policies of the Government of Nepal helped to minimise the impact of COVID-pandemic on school education and, at the same time, allowed the government to adapt school education. The policy interventions were helpful in reinstating school education through alternative mediums and mobilising the strength of local stakeholders, such as local governments and schools. More importantly, the initiation of alternative modes of education, such as virtual classes, has tended to make education system resilient during the crisis (Pal et al., 2021). These policy approaches have not only protected the right of children to receive education but local school stakeholders have also realised their potential. Table 2 presents a summary of findings, especially with respect to how the newly instituted policies in the COVID context helped to strengthen local management in school education.

**Table 2.** Summary of the education policies implemented during the 2020 COVID-pandemic.

S.n	Educational policies	Approved date	Interventions	In education
1	COVID-19 education cluster contingency plan, 2020	May 2020	Envisaged collaborative support amongst all tiers of governments to handle the pandemic situation	Policy
2	Alternative System’s learning Facilitation Guidelines, 2020	May 2020	Responsibilities passed to local governments and schools to begin alternative teaching- learning	Pedagogy
	Student learning facilitation Guidelines, 2020 [revision of the alternative System’s learning Facilitation Guidelines, 2020]	September 2020		
3	Secondary education Examination’s student assessment, result publication, and verification regulations 2020	June 2020	Authorised schools to evaluate the SEE enrolled students	Assessment
4	School level curriculum adjustment framework, 2020	September 2020	Authorised local governments and schools to implement revised curriculum and appropriate evaluation practice	Curriculum and assessment
5	Education related emergency guidelines, 2020	September 2020	Tasks assigned to local governments and schools to keep an eye on vulnerable students, continue teaching-learning through alternative means, arrange online resources in schools, and mobilise teachers	Governance and management
6	COVID related school operation guidelines, 2020	November 2020	Delegated school opening authority to local governments	Governance and management

## Acknowledgment of Localism in School Education

The policy additions in the Nepali education sector due to the COVID-pandemic is a paradigmatic shift in Nepal’s educational development. Before the COVID-pandemic, there were several efforts made to meaningfully decentralise school education to the local level. Unfortunately, the decentralisation process in Nepal was not in full operation as envisaged by the 2015 Constitution (United Nations Children’s Fund, 2021c). The decentralisation of school management has always been a slow process in Nepal as it seems the central bureaucracy was largely uninterested in the process (Hamal, 2020; Khanal, 2010). The COVID-pandemic has exerted pressure on the meaningful decentralisation in school governance in Nepal. This change is expected to happen at the time when all global indicators of development are sharply declining (United Nations Development Programme, 2022). The strength of local governments and schools has been highly acknowledged by the Central Government. Principally, Nepal Government has acknowledged the essence of Localism which is a bottom-up approach to people’s development and self-governance (Chaudhary, 2019b). Localism advocates collaborative ideas between all stakeholders to make a society’s development happen (Mohan and Stokke, 2000; Kurland et al., 2012; Mansuri and Rao, 2012). The Government has highly considered the strength of local governments, schools, and communities. Similar to the claim of Karki (2020) about the COVID-pandemic and its opportunities for local governments, the clear guidelines from the Central Government of Nepal helped local governments to actively and effectively engage in school education. The six-policies interventions have established and institutionalised the strength of local governance in school education in Nepal.

First, the COVID-19 Education Cluster Contingency Plan was as an immediate action taken by the Government of Nepal to take control of school education. The Central government asked for a collaborative approach with all provincial and local governments to handle the impact of COVID on education. Second, the Alternative System’s Learning Facilitation Guidelines and its amendment includes the roles and responsibilities of local governments to begin teaching-learning through alternative means. Third, the Secondary Education Examination’s Student Assessment, Result Publication, and Verification Regulations delegated responsibility to schools to evaluate the final exams of Grade 10 students and provide the marksheets for approval by the Nepal National Examination Board (NEB). Previously, this was the direct responsibility of the NEB. Fourth, the School Level Curriculum Adjustment Framework authorised the schools and local governments to implement the shortened curriculum in the COVID context and adapt student’s evaluation process, as necessary. The revisions in the curriculum were made to complete the school courses in the same academic year of 2020 [2076/7 B.S.]. Fifth, the Education Related Emergency Guidelines provided clear roadmaps to schools and local governments to help students with their enrolments, teaching-learning, textbooks, and alternative learnings. Sixth, the COVID Related School Operation Guidelines was a step forward because it authorised local governments to decide when to resume face-to-face teaching in schools.

Above mentioned six policy interventions helped to sustain schooling education and take care of the well-being of students during the COVID in Nepal. These policies have acknowledged that schools and local governments have been responsible for planning and implementing their local response to adapt school education during the COVID pandemic (Nepal Ministry of Education, 2021). The delegation of school-related authorities by the Central governments to the local governments is the adoption of horizontal governance, which encompasses multiple stakeholders in the policy planning and implementation process (Halligan, 2020; Keuffer and Horber-Papazian, 2020). The horizontal exercise of power supports the theory of localism in which the roles of bottom stakeholders are prioritised (Kurland et al., 2012). Mansuri and Rao (2012 suggested that the development process becomes effective and result-oriented if both central state and local initiatives participate. Rizvi and Lingard (2009) argued that the actual devolution of power only happens when people have the right to make decisions and control. In our research’s context, the local governments represent the people, and we claim that the education-related power has been devolved to these governments representing people at the community level.

Besides, the role of local governments was influential in winning communities' trust to continue children’s learning. Dabrowski et al. (2022) cited an example of Bangladesh to show how the community’s effort helped minimise the dropout rates and bring students back to school after COVID. In the Nepali context, there are examples to show the roles of the SMCs in facilitating the teaching-learning activities by teachers by forming Village Learning Groups during the pandemic. The alternative teachings were more effective in communities with active SMCs (United Nations Children’s Fund, 2021c). There was a provision of *Tole Sikshya* (Education in Community) as one of the alternative teaching-learning in communities. Messaging through community members was a medium for teachers to connect with students (United Nations Children’s Fund, 2021a). Families had concerns about both the well-being and learning loss of students. After the central government issued the 2020 COVID-Related School Operation Guidelines, parents and communities gave moral support to schools in resuming physical classes. They began sending their children by adopting necessary health measures such as facemasks, asking them to wash their hands repeatedly, and maintaining social distance from colleagues. The encouragement of parents to send their children to schools after COVID is substantiated by research conducted by UNICEF, which mentioned that 89% of Nepali parents were willing to do this (United Nations Children’s Fund, 2021a). Likewise, United World School, a school run by a Non-Government Organisation to support Nepal’s education, conducted different campaigns to sustain students' learning with the engagement of community teachers. This includes preparing awareness material and disseminating regarding COVID’s safety, engaging students in regular learning activities such as story writing, art competition, and poem competition, and involving students in learning activities through radios (Jha, 2020, April 20). COVID situation established the horizontal exercise of power through local governments, schools, and communities to continue schooling education.

However, from a macro-level policy perspective, local governments were proactive in handling the COVID-pandemic situation to sustain school education, and there was the disengaging role of communities. There are some reasons behind this claim. First, local governance in schooling education is a new concept initiated after local governments formed through the 2017 election in Nepal. Until 2022, there is no Federal Education Act to institutionalise decentralised educational governance. Nepal is in the Federal transition stage, where a large political authority has been devolved to local governments. This stage demands the institutional capacity enhancement of local governments to govern schools, institutionalise decentralisation, and promote quality education (Nepal Ministry of Education, 2021). The COVID situation in 2020 and 2021 engaged the Central Government to frame education-related policies and local governments to implement these. Local governments played a vital role in sustaining schooling education. However, the roles of SMCs were not influential during the pandemic. This is due to the lack of administrative and political authority for the SMCs. Another reason is the unclear roles of local school stakeholders due to the federal transition. Second, different educational policy documents after 2015 suggest SMCs as a supportive institution to local governments in managing school education (see Nepal Ministry of Education, 2019b; Nepal Ministry of Education, 2016; Nepal Ministry of Education, 2019c). This suggests a hierarchical mindset in the government’s bureaucracy, which is still reluctant to power-sharing with beneficiaries at the implementation level (Acharya and Scott, 2022; Acharya, 2021). Third, local governments want a significant power status in schools which has minimised the roles of communities. During the pandemic, local governments were more accountable to the Central Government because local governments rely on financial support, teachers' deployment, educational policies, and technical training such as curriculum-making with the Central Government (Dhungana et al., 2021). This power role of the Central government and the COVID-related response of the local governments delimited the roles of communities in schools during the pandemic. Fourth, the Central Government issued a one-door tackling policy to handle the COVID situation and provide relief to citizens (Basnet et al., 2021; Shrestha et al., 2021). In this situation, government mechanisms such as local governments were only reliable and trustworthy enough to oversee schooling education at the community level. These scenarios suggest that related educational authorities have been delegated from the Central government to the local governments; however, communities are still perceived as service users instead of stakeholders in the educational development process.

Despite the abovementioned initiatives to handle COVID-pandemic in Nepal, there are some dark features of the pandemic in the education system. COVID-19 has retarded development growth (Nepal National Planning Commission, 2021b). It has negatively impacted on Nepal graduating from Least Developed Country status to Developing Country by 2026, and to achieve the Sustainable Development Goals by 2030 (Nepal National Planning Commission, 2020b; United Nations, 2021). There is no Federal Education Act in Nepal which could have clearer roles, responsibilities, and authorities on school education amongst of all tiers of governments. This act was expected to be prepared soon after promulgation of the 2015 Constitution that politically restructured the country into federal and local units (Dhungana et al., 2021). The 2020 15th Plan of Nepal has no specific measures to sustain the school education during pandemic times (Nepal National Planning Commission, 2020a). Schools in Nepal lack ICT infrastructures to continue teaching-learning in pandemic situation (Adhikari, 2019). During the pandemic, poor children were adversely affected by not having access to the internet to continue their online classes (Nepal National Planning Commission, 2021a). Alternative education through the internet, radio and television were fruitful to students with better access to these facilities. Only 56% of Nepalis have access to the internet. There is a situation of a digital divide in education between rich and poor students in Nepal (Dhungana et al., 2020; Dawadi et al., 2020). The impact of the educational crisis due to the pandemic is sure to have long lasting effects on young children and a country’s growth (United Nations Development Programme, 2022). However, in terms of education-related power delegation to local governments in Nepal, COVID-19 has inadvertently allowed an important breakthrough. The pandemic has become a catalyst to develop inter-governmental relationships amongst all tiers of governments in the country (United Nations Children’s Fund, 2021c), including in education reform. The 2020 15th Plan of Nepal creates an opportunity to reform school education through the management of all levels of governments with their gradual capacity enhancements (Nepal National Planning Commission, 2020a). Nepal has adopted the collaboration between the national and subnational governments delegating political authorities to respond the pandemic.

One important commonality found in recent innovations in the educational policies in Nepal, is the acknowledgment of localism. Localism favours local and acknowledges its engagements in decentralised development process (Madanipour and Davoudi, 2015). The role of the Central Government was of course crucial in preparing the guidelines for the way out of the COVID pandemic’s effect on education. Local governments and schools were at forefront of following the advice of the Central government and helping children to continue their education; otherwise, there could be an enormous dropout of young people from school (Dawadi et al., 2020). The 2021 Nepal Education Sector Analysis report mentions that these recent policies were developed to respond the educational crisis in COVID-pandemic. These policies delegate administrative and political authority to local governments in responding to the pandemic locally (Nepal Ministry of Education, Science and Technology, 2021). These additions of educational policies in the COVID context in Nepal are likely to institutionalise the supremacy of local governance in the meaningful development of the education sector. Thus, the centrality of local governance in implementing educational plans, policies, and interventions in schools in Nepal has been highly acknowledged after the COVID-pandemic.

## Conclusion

The roles, responsibilities, and authorities of local governments regarding school education were mentioned in the 2015 Constitution and the 2017 Local Government Operation Act of Nepal. However, it could have been difficult to institutionalise these roles quickly if there was no COVID-pandemic. The pandemic brought on an opportunity to institutionalise local governance in school education. It has brought an opportunity to decentralise school governance at local levels in the country. During the first wave of the pandemic, six educational plans, guidelines, and regulations were prepared to cater to the needs of students, teachers, schools, local governments, and other stakeholders. All these new policies have centralised the roles of local governments and schools in dealing with the COVID-pandemic at the forefront. These interventions have addressed the concerns of pedagogy, assessment, curriculum, governance and management, and educational policies in the COVID context in Nepal. The changes induced due to the pandemic in Nepali school governance should be institutionalised by the Central Government by sincerely adopting the agreed legislation, which mobilises the strength of local governments. The distribution of education-related powers to the local governments strengthens public education through local and mutual efforts, which is also a practice of horizontal governance. Communities should be considered a collaborator by the local and central governments to help schools during a such pandemic. Overall, the COVID-19 pandemic has reinstated localism as a vital component in school governance in Nepal.

## Notes

1 Approved by the Government of Nepal

2 Delta

3 Omicron

4 B.S.: Bikram Sambat is a Nepali calendar

5 Belongs to Local Government Offices
